# Double Lateral Osteotomy: Not Only the Correction of Crooked Noses but a Relevant Aesthetical Refinement in Structural Rhinoplasty

**DOI:** 10.3390/jpm13111619

**Published:** 2023-11-19

**Authors:** Roberto Bracaglia, Maria Servillo, Regina Fortunato, Anna Amelia Caretto, Stefano Gentileschi

**Affiliations:** 1 Department of Plastic Surgery, Bracaglia Aesthetic Center, Villa Stuart Clinic, Eurosanità s.p.a., Via Trionfale 5952, 00135 Rome, Italy; 2 Department of Plastic Surgery, Department of Women’s, Children’s and Public Health Sciences, Fondazione Policlinico Universitario A. Gemelli IRCCS Roma, L. go Agostino Gemelli 1, 00168 Rome, Italystefanogentileschi@gmail.com (S.G.); 3 Dipartimento di Medicina e Chirurgia Traslazionale, Facoltà di Medicina e Chirurgia, Università Cattolica del Sacro Cuore, 00168 Rome, Italy

**Keywords:** deviated nose, FACE-Q, nasal width, nose, osteotomy, rhinoplasty

## Abstract

Background: Osteotomy represents a crucial step in structural rhinoplasty; however, there is not a unique approach accepted. Double lateral osteotomy has proven to be effective in the long-term correction of a deviated nose. In this series, we evaluated its aesthetic value also in non-deviated cases. Materials and Methods: 864 patients who underwent primary structural rhinoplasty from 2012 to 2020 were divided into four groups. Group A and B included patients with a crooked nose treated with asymmetrical double osteotomy and bilateral double osteotomy, respectively. Patients who did not present nasal deviation were divided into group C, including cases treated with bilateral single osteotomy, and group D, including patients who underwent bilateral double osteotomy. Postoperative evaluations were performed by three independent plastic surgeons blinded to the surgical technique. Patient’s satisfaction was assessed through the FACE-Q rhinoplasty module. Results: FACE-Q scores reported a satisfaction rate higher than 30% for every item in all groups; however, group B and group D showed statistically higher satisfaction (*p* < 0.01). According to the evaluations performed by physicians, group B and group D showed the most satisfactory outcomes (*p* < 0.01). Conclusions: bilateral double osteotomies represent a significant aesthetic refinement in structural rhinoplasty, not only in crooked noses but also in non-deviated cases, since the reduction in the width of the nose is an aesthetical aspect very appreciated by patients.

## 1. Introduction

Lateral osteotomy is a mainstay of structural rhinoplasty as it allows different concerns to be resolved: closure of the open roof after dorsal hump removal, narrowing of the nasal pyramid, straightening of convex nasal bones and correction of the asymmetry of nasal walls [1,2]. In 2004 [3], authors introduced the concept of double lateral osteotomy, a technique that has proven to be very effective in crooked noses, in which it allows the asymmetrical growth of the maxillary processes to be corrected. In that work [3], authors proposed to perform a unilateral double osteotomy on the contralateral side of the deviation, where the maxillary process was longer and more convex, and a single osteotomy on the shorter and more concave side. In this way, the double osteotomy allowed the surgeon not only to move medially the bony fragments, closing the bony roof, but even to reshape the convexity of the longer nasal wall, changing the outline of the more developed side. Since then, the extensive experience of the authors in rhinoplasty in crooked noses [3,4,5] has led them to perform double lateral osteotomy bilaterally, also in the more concave side, to reshape even the curvature of the shorter nasal wall. Moreover, bilateral double osteotomy proved to be very effective in reducing bony nasal width, creating a more attractive ratio between malar and nose extent, not only in very wide noses but also in most non-deviated cases. Patient satisfaction is an essential outcome measure after a rhinoplasty; however, it is sometimes overlooked and not always in accordance with surgeons’ perspective [6,7]. The aim of this study is to evaluate aesthetic outcomes and patient satisfaction with the use of bilateral double osteotomy in primary rhinoplasty in deviated and non-deviated noses.

## 2. Materials and Methods

This study included 864 patients who underwent primary structural rhino-septoplasty from 2012 to 2020, performed by the same surgical team, with at least one year of follow up. The exclusion criteria were as follows: revision rhinoplasty, complications following rhinoplasty, presence of congenital malformation, uncontrolled systemic diseases, major psychological problems, age younger than 16 years and less than one year of follow up. This study was conducted in accordance with the Declaration of Helsinki guidelines, and written informed consent was obtained from all patients. The demographic and clinical data of all patients were collected from clinical records and during follow up by means of a questionnaire, and a complete physical examination was performed. Standard preoperative photos (8 views) were taken of all participants with a Nikon camera using a 70 mm macro lens (105 mm effective focal length). The frontal images were taken in the Frankfurt horizontal plane. The same standard pictures were obtained for comparison at 6 and 12 months of follow up, and then every 5 years thereafter. The patients were divided into 4 groups. The first group included patients with a crooked nose corrected by rhinoplasty with asymmetric double osteotomy (group A); group B included patients with a crooked nose who underwent rhinoplasty with bilateral double osteotomy. Patients without nasal deviation were divided into group C, which included cases treated with bilateral single osteotomy, and group D, which included patients who underwent bilateral double osteotomy. Postoperative evaluations of the outcomes were performed by 3 independent plastic surgeons who were blinded to the surgical technique. They evaluated preoperative and postoperative photographs in all of the 8 views, and aesthetic outcome assessments were expressed though a Visual Analogue Scale (VAS scale) (from 0 to 5 points) which considered global aesthetic outcome, frontal view, profile view, basal view and scarring. VAS scale is a psychometric response scale used to measure subjective characteristics or attitudes which has been largely used for a multitude of social science investigations and research [8]. Patient satisfaction was assessed through the Italian version of the FACE-Q rhinoplasty module [9] that we provided electronically to each patient at least 1 year after the surgical procedure. The FACE-Q rhinoplasty module represents one of the most used and reliable patient-reported outcome (PRO) measures, providing a short, easy-to-complete, valid and responsive tool to evaluate patient satisfaction. The Italian linguistic translation process, validated in 2018, led to a conceptually equivalent Italian version of the FACE-Q Satisfaction with a nose scale [9]. Data were analyzed with SPSS 21 for Windows (SPSS Inc., Chicago, IL, USA). Measurements are reported as absolute number and percentage for categorical variables and mean ± standard deviation (SD) for quantitative variables. The Fisher exact test was used to compare the categorical data between two groups. *p* < 0.05 was considered significant.

### Surgical Technique

**Figure 1:**
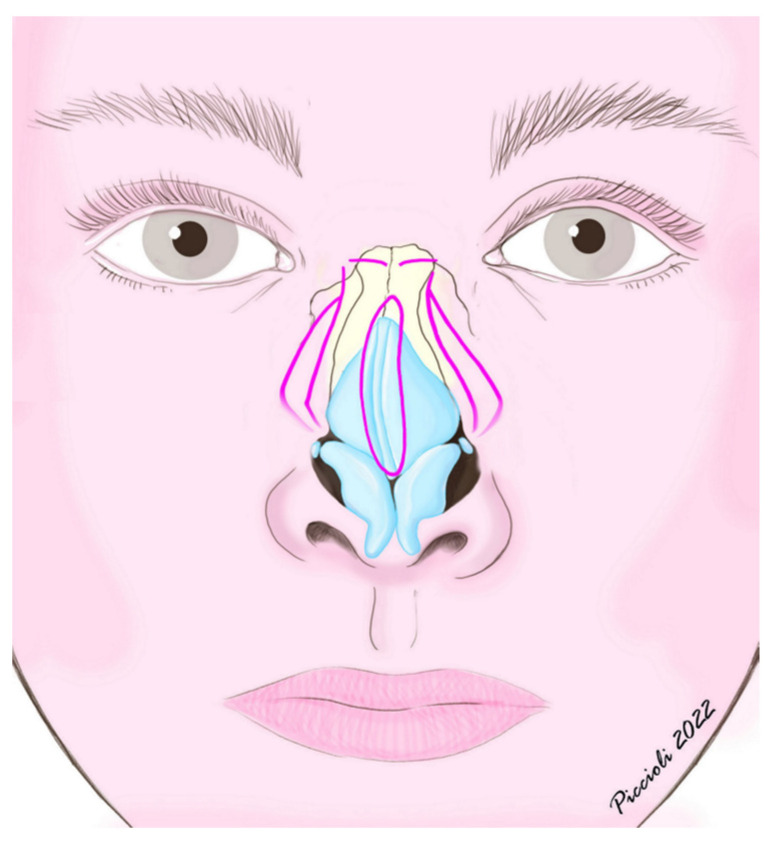
Schematic representation of bilateral double osteotomy, frontal view.

All procedures were performed under general anesthesia, using the closed or the open-tip approach [10]. Osteotomies are generally performed during the final stages of the operation, after the dorsal–nasal height, septum and tip have been addressed, to avoid the eventual increase in intraoperative edema which could reduce visual evaluations. We performed all of the osteotomies, two lateral and one medial for each side, through an endonasal continuous approach. The first osteotomy was the more medially located lateral one which was started at the pyriform crista, just above and anterior to the attachment of the inferior turbinate. It was started slightly more anterosuperior than the most posterior point of the piriform aperture edge, to preserve Webster’s triangle, a triangular portion of maxillary frontal process near the internal valve area [11]. The osteotomy was then conducted with a high-to-low pattern and, depending on the side, the authors followed the line of greater convexity or concavity of the bony nasal wall, guiding the osteotome to the level of the intercanthal line, bilaterally. After the completion of the first lateral osteotomy on each side, a bilateral medial osteotomy was always performed through an endonasal approach: from the superior nasal spine, the medial osteotome was conducted with a superior oblique pattern to the intercanthal line, leaving a few millimeters of intact bone from the lateral osteotomy line, thus allowing an easy digital infracture and closure of the open roof. After the closure of the nasal vault the second lateral osteotomy was performed, parallel but more lateral than the first one, starting from the same level in the piriform aperture and then moving laterally toward the maxilla with a high-low-high pattern, ending by joining with the first one below the intercanthal line (Figure 1). The distance between the two osteotomies was chosen according to the degree of narrowing of the nose needed, the asymmetry of the nasal bony structure and the protrusion of the maxillary process. We did not elevate the periosteum, since the overlying soft tissue and muscles, such as transverse fibers of nasalis muscle and levator labii superioris alaeque muscle, as well as the integrity of the underlying mucosa, constitute a useful support for the bone fragments, increasing their stability and decreasing the incidence of air flow obstruction and contour irregularities. The choice of the right osteotome was crucial in order to minimize the trauma of soft tissues [1,12,13]. The authors routinely employed 3 mm guarded curved osteotomes which allowed them to perform both levels of osteotomies, even the lowest one, where the maxillary bone was thicker. Other paramount aspects of surgical technique, especially for crooked noses, are asymmetric hump removal, septoplasty with complete correction of lamina quadrangularis and vomer, stabilization of the septum with bilateral spreader grafts and regularization of the inferior nasal spine. While respecting the keystone area, septoplasty was performed through a complete dissection in the subperichondial/subperiosteal plane of the perpendicular plate of the ethmoid, lamina quadrangularis and vomer, followed by resection of the lower obstructing/deviated portions and partial chondrotomies of the quadrangular cartilage to reduce the elastic deviation forces. Generally, it was performed after hump reduction and before addressing the tip and nasal bones. The amount of non-deviated septum left depended on the need for cartilage grafts; however, at least 1 cm dorsal–caudal quadrangular cartilage L-strut was always left. These technical steps allowed an effective symmetrization approach of the frontal processes of the maxillary bones. After the procedure, the nose was packed for one day, and the patient wore a dorsal splint for one week.

## 3. Results

A total of 864 patients were enrolled in the study, of whom 353 (40.9%) were male and 511 (59.1%) were female. The mean age of the patients at the time of operation was 33.4 years (range, 18–62 years). The mean follow-up time was 3.7 years (range 1–9 years). Group A included 171 patients; Group B 144 patients. Non-crooked noses were divided into group C, which comprised 291 cases, and group D, with 258 patients. FACE-Q scores reported a satisfaction rate higher than 30% for every item in all groups; however, group B and group D showed statistically higher satisfaction than group A and C, in particular as regards the width of the nose (*p* < 0.00001), how the nose suited the face (*p* < 0.00001), the overall size of the nose (*p* < 0.00001) and how the nose looks in photographs (*p* < 0.00001) (Table 1). According to the evaluations performed by the three physicians, group B and group D showed the most satisfactory outcomes, which were statistically significant regarding frontal view, basal view, feminine/masculine shape and global aesthetic outcome (*p* < 0.01) (Table 2). A total of 25 patients required a secondary procedure for osteotomy revision, of which 8 patients from group A for incomplete bone straightening and mobilization, 4 patients from group B for palpable irregularities, 12 patients from group C for palpable irregularities and insufficient bone mobilization and 1 patient from group D for palpable irregularities. All of the cases were corrected at 6 months postoperatively through additional internal osteotomy to provide better bone mobilization or directly minimize bone irregularities.

## 4. Discussion

**Figure 2:**
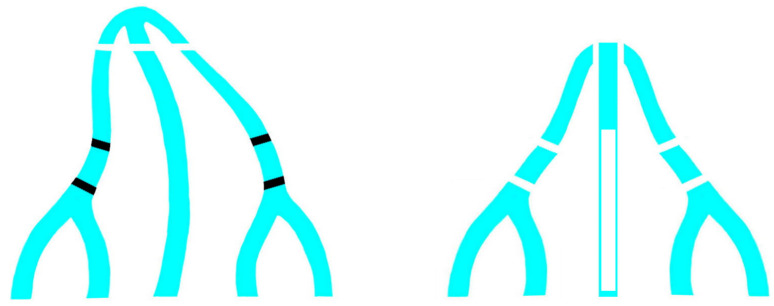
Schematic representation of bilateral double osteotomy in deviated nose, basal view. Bilateral double osteotomies are useful to reshape both nasal walls; in the crooked nose, it helps to symmetrize nasal bones with different shapes and curvatures. In these cases, asymmetrical hump removal and septoplasty with resection of the deviated portions of the lower cartilage and bony septum (white rectangular resection in the septum) and partial chondrotomies are other paramount aspects to achieve a stable correction.

Osteotomies represent a crucial step in structural rhinoplasty due to their prominent role in the achievement of ideal functional and aesthetic results. Despite the growth of instruments used and techniques of osteotomy, there is not any unique accepted approach. Some of the more critical aspects are represented by the level and the direction of the osteotomy line, the possible comminution of the fracture, the surgical trauma to soft and bony tissues and the degree of the reduction in the anterior part of the nasal cavity, which may determine airflow obstruction [11,14,15]. Monolateral double osteotomy has been employed for a long time by the authors in the management of severe deviation of the nasal pyramid [3]; however, their wide experience in crooked noses led them to consider bilateral double osteotomy as a more effective way to reshape the curvature of both nasal walls. Similar to the more developed and convex side, the curvature of the more concave nasal wall must be corrected and reshaped, too. Bilateral double osteotomy, in association with asymmetrical hump removal, allows surgeons to symmetrize nasal walls not only in terms of size but also in terms of shape and curvature (Figure 2).

**Figure 3:**
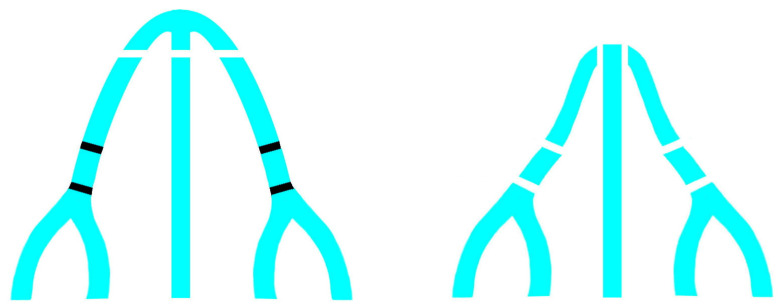
Schematic representation of bilateral double osteotomy in a non-deviated nose, basal view. In these cases, this technique creates a concave configuration of the frontal process of the maxilla which produces a narrower looking nose with deeper shadowing of the lateral contours.

Moreover, bilateral double osteotomy simplifies the closure of open nasal roof, allowing for an easier medial movement of the bony fragments. The creation of two levels of movement decreases tension and achieves a more stable closure of the open roof. This was recognized also by Tabrizi et al. who showed the long-term effectiveness of double lateral osteotomy in the correction of crooked noses compared to asymmetric dorsal hump reduction and ascribed it to a more reliable relieving of tension vectors in the deviated nose [15]. Parkes et al. illustrated a technique of double lateral osteotomy in which they performed the second osteotomy at a higher level, along the path of less resistance, anteriorly and superiorly to the thick part of the maxillary process inside the nasal wall [16]. The aim of this higher osteotomy should be to obtain a dorsal fragment primarily consisting of the nasal bone to allow an easier closure of the nasal roof without medial displacement of the inferolateral portion of the maxillary process, which may cause narrowing of the nasal airway. In our study, the second lateral osteotomy was performed at a lower level than the first one for several reasons. Firstly, in this way, the medial movement of the fragments does not involve only the more dorsal part of the nasal wall but also part of the maxillary process, avoiding the excessive narrowing of the nose and the postoperative aspect of a “surgical nose”. The movement of the inferolateral portion of the maxillary process did not cause any functional problem thanks to the preservation of the Webster’s triangle, a triangular area of the caudal aspect of the frontal process of the maxilla abutting the piriform aperture, which prevents the collapse of the internal nasal valve [11,17]. Moreover, a thicker bone easily resists comminution of the fracture. Regarding the order in which the osteotomies are performed, some authors propose to perform medial osteotomies after hump resection and before lateral ostetomies [1,18,19,20,21], motivating it with a greater stability of the nasal bones during fracture and the possibility of reducing the width of the nasal dorsum even with only these medial fractures. Otherwise, our experience led us to believe that lateral osteotomies and subsequent medial superior oblique osteotomies give the more stable and predictable results in terms of medialization of bones and closure of the open roof. We agree with Block et al. about the sequence [22], in which the lateral osteotomies come first followed by the medial ones, that they appropriately call “medial-to-lateral”, since it plays an important role in achieving a precise and predictable fracture and subsequent bone medialization. As they note, the precise and stable execution of the medial osteotomy depends primarily on the integrity of the keystone area, rather than on performing it before the lateral osteotomy. We also agree with their vision that in the correction of crooked noses, minor maneuvers and a limited approach will inevitably result in under correction and/or relapse, and only an extensive reconstructive procedure will consistently result in a satisfactory outcome [22]. Westreich and Lawson recognized the aesthetic advantage of a double lateral osteotomy in many indications: narrowing of the wide nose, reshaping the convex nasal pyramid, recreating symmetry in the traumatic and deviated nose, reducing the wide and prominent maxillary process and eliminating ledges in primary and revision rhinoplasty [23]. Similar to our findings, they believe that a single osteotomy may produce closure of the nasal roof, but it leaves a wide and unattractive nose with limited shadowing on the nasal sidewall. Creating a concave configuration of the frontal process of the maxilla with double osteotomies produces a narrower looking nose with deeper shadowing of the lateral contours (Figure 3).

**Figure 4:**
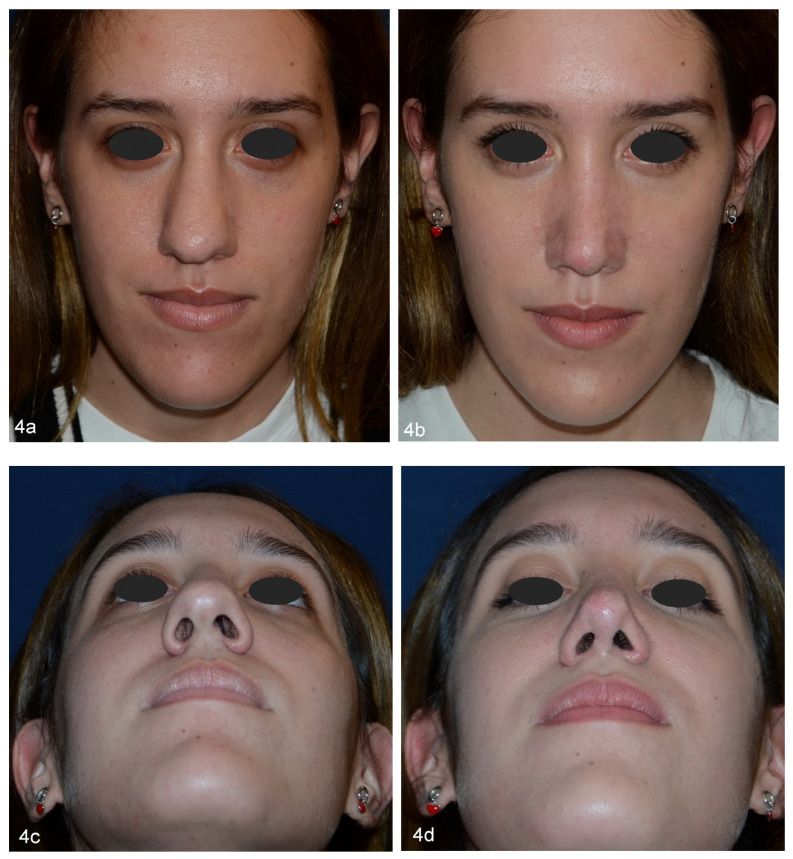
Preoperative (**a**–**c**) and 3-year postoperative (**b**–**d**) views of a 21-year-old patient of group D. In non-deviated noses, double lateral osteotomies allow the surgeon to achieve a narrower aspect of the nasal pyramid, creating a more attractive ratio between malar and nose extent.

**Figure 5:**
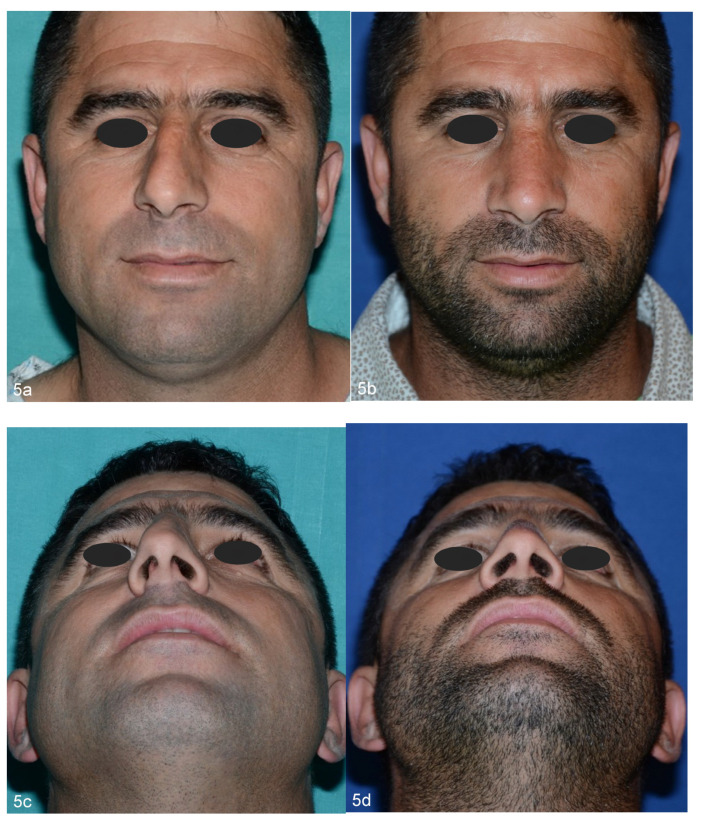
Preoperative (**a**–**c**) and 5-year postoperative (**b**–**d**) views of a 45-year-old patient of group B. In crooked noses, bilateral double osteotomies, in association with asymmetrical hump removal and septoplasty, allow the surgeon to symmetrize both nasal walls not only in terms of size but also in terms of shape and curvature.

This is in accordance with our FACE-Q scores which showed higher satisfaction of patients who underwent double osteotomies, particularly with regards to the width of the nose and how the nose suited the face. Moreover, Westreich and Lawson underlined that a double lateral osteotomy prevents the occurrence of palpable or visible ledges of maxillary bone which could result from a conventional single lateral osteotomy when there is a thick and prominent attachment of the frontal process of the maxilla to the nasal bones. Both reviewers and patients, through the questionnaires, particularly appreciated the aesthetic outcome in terms of nose width, how the nose suited the face, the overall size of the nose and how the nose looks in photographs. We believe that this is due to a more favorable ratio between the width of the nose and the face, given the decrease in the space of the nasal bone base in favor of a greater malar extension. With a single osteotomy, it is possible to close the open roof, but the malar extent does not increase as it is still limited by the bulging of the frontal processes of the maxilla. With double osteotomies, these structures instead contribute to the bone base of the nose, visually enlarging the malar space and giving the nose an even more natural and less “surgical” appearance (Figure 4a–d and Figure 5a–d).

This could also be correlated with the neoclassical canon according to which, especially in the female sex, the width of the nose should be a quarter of the width of the face [24]. It should be noted that the neoclassical canon refers to the width of the alar base but other studies have reported that the width of the bony base should be equal to 75% to 80% of the alar base width or the intercanthal distance [25]. In 2009, Torsello et al. evaluated photographs of 50 Caucasian female models selected for an important beauty contest and they observed that the nasofacial proportion demonstrated a tendency for the facial width to be equal to (24%) or higher (52%) than four times the width of the nose [26]. On average, nose width was 1.2 mm smaller than ¼ of face width, and this difference was statistically significant (*p* < 0.05). Although these aesthetic canons were generally applied to the female sex, in our series, we did not observe statistical differences between the sexes.

Revision cases (3%) were limited to incomplete bone mobilization and irregularities, easily correctable with additional osteotomies, while the collapse of bony fragments towards the nasal cavity was never observed, most likely because the periosteum was not elevated and because the frontal process of the maxilla kept tight connection with muscles of the Superficial Musculo-Aponeurotic System (SMAS), particularly the transverse portion of the nasalis muscle and the angular portion of the levator labii superioris muscle. No mucosal damage could be detected during the follow up by speculum examination. As we mentioned, all of the cases in this study were performed with the open-tip approach. We believe that the treatment of the dorsum in a closed approach has the advantage of allowing a direct evaluation of the relationship between the reduction in the bony-cartilaginous framework and the overlying soft tissues. In this context, performing lateral osteotomies with an endonasal approach, associated with a very limited undermining of soft tissues on the nasal dorsum, has the advantage of providing a support to bony fragments, thus reducing the risk of internal collapse. On the other hand, open approaches, in particular, with piezoelectric instruments, have the undeniable advantage of wide exposure which allows surgeons to perform precise osteotomies under direct visual control and also ostectomies and the so-called “rhinosculpture” [27]. In this study, we did not use piezotome; however, it could be possible to perform double lateral osteotomies with piezotome, keeping the integrity of internal mucosa to avoid collapse of the bony fragments. Good indications for the use of piezotome could be cases with significant bone curvature, where the use of piezotome could allow a real osteoplasty with reduction in bone thickness, and cases with short, osteoporotic or brittle nasal bones, where the piezotome could reduce the risk of comminution of fragments [27,28]. In the treatment of these complex cases with conventional endonasal osteotomies, we would like to stress even more the importance of avoiding damage to the soft tissue above and below the osteotomy lines; the osteotome should progress by cutting the bone in a tunnel under the periosteum and over the mucosa, leaving these tissues as intact as possible. Thus, in the occurrence of comminution of bones, we proceeded with closed reduction and the positioning of external splints; however, we principally rely on the mucosal and soft tissue integrity for proper bone healing. In these cases, external splints, which we generally keep in place for 5 days, are kept for 10 days. All of the patients were routinely informed about the criticisms regarding bone remodeling and the importance to wear the external splint for a period variable from 5 to 10 days. Accordingly, all of the patients were instructed that nose blowing is forbidden for at least 14 days [29]. Limitations of the study are represented by the monocentric design and the relatively shorter follow up in the groups of patients who underwent bilateral double osteotomy.

## 5. Conclusions

Bilateral double osteotomies represent a significant aesthetic refinement in structural rhinoplasty, not only in crooked noses but also in non-deviated nasal pyramids, since the reduction in the width of the nose in favor of a wider malar space is a very appreciated aspect by patients in the overall aesthetics of the face.

## Figures and Tables

**Table 1:** Face-Q satisfaction with the nose postoperative module ^1^.

Face-Q Items	Group A (171)	Group B (144)	Group C (291)	Group D (258)	*p* A-B	*p* C-D
The width of your nose at the bottom?	120 (70%)	134 (93%)	195 (67%)	250 (97%)	*p* < 0.01	*p* < 0.01
The length of your nose?	105 (61%)	139 (96%)	174 (60%)	233 (90%)	*p* < 0.01	*p* < 0.01
How the bridge of your nose looks (where glasses sit)?	95 (55%)	140 (97%)	165 (57%)	250 (97%)	*p* < 0.01	*p* < 0.01
How well your nose suits your face?	120 (70%)	135 (94%)	204 (70%)	254 (98%)	*p* < 0.01	*p* < 0.01
How straight your nose looks?	95 (55%)	134 (93%)	200 (69%)	233 (90%)	*p* < 0.01	*p* < 0.01
The overall size of your nose?	120 (70%)	140 (97%)	204 (70%)	250 (97%)	*p* < 0.01	*p* < 0.01
How your nose looks in photographs?	104 (61%)	140 (97%)	200 (69%)	250 (97%)	*p* < 0.01	*p* < 0.01
How your nose looks from every angle?	105 (61%)	135 (94%)	204 (70%)	233 (90%)	*p* < 0.01	*p* < 0.01

^1^ Raw score ≥ 3, Fischer exact test, *p* < 0.05.

**Table 2:** Surgeon reviewers VAS scale ^1^.

Items Evaluated	Group A (171)	Group B (144)	Group C (291)	Group D (258)	*p* A-B	*p* C-D
Global cosmetic outcome (mean)	3.0	4.6	3.6	4.8	*p* < 0.01	*p* < 0.01
Scarring (mean)	3.9	4.8	4.4	4.8	0.1	0.1
Profile view (mean)	4.4	4.6	4.4	4.6	0.3	0.3
Frontal view (mean)	2.6	4.6	2.8	4.8	*p* < 0.01	*p* < 0.01
Basal view (mean)	3.2	4.4	3.4	4.8	*p* < 0.01	*p* < 0.01
Feminine/Masculine shape (mean)	3.3	4.4	3.8	4.8	*p* < 0.01	*p* < 0.01

^1^ From 0 to 5 points. Fisher exact test, *p* < 0.05.

## Data Availability

Data are contained within the article.
